# Cell fusion as a link between the SARS-CoV-2 spike protein, COVID-19 complications, and vaccine side effects

**DOI:** 10.18632/oncotarget.28088

**Published:** 2021-12-07

**Authors:** Yuri Lazebnik

**Affiliations:** ^1^Lerna Consulting, New Haven, CT 06511, USA

**Keywords:** cell fusion, thrombosis, neuropathy, cancer, vaccines

## Abstract

A distinctive feature of the SARS-CoV-2 spike protein is its ability to efficiently fuse cells, thus producing syncytia found in COVID-19 patients. This commentary proposes how this ability enables spike to cause COVID-19 complications as well as side effects of COVID-19 vaccines, and suggests how these effects can be prevented.

## INTRODUCTION

A hallmark of severe COVID-19 is the abundance of syncytia, the products of fusion between two or more cells in the lungs of patients [1–3]. These syncytia have been attributed to the ability of spike, a protein encoded by SARS-CoV-2, to fuse cells to each other, and prompted a search for drugs that could prevent this cell fusion. Recently, Braga and colleagues [3] identified a set of already approved drugs that prevent spike-induced cell fusion and inhibit TMEM16F, a protein that has two activities [4]. One, a calcium-activated ion channel, regulates chloride secretion, while the other, a lipid scramblase, relocates phosphatidylserine (PS) to the cell surface in a process known as PS externalization.

PS externalization is required for cell fusion in many systems, [5, 6] which explains why inhibiting a scramblase prevents the formation of spike-induced syncytia. However, Braga and colleagues have concluded that although PS externalization “is required for plasma membrane fusion, chloride secretion might have relevance in COVID-19 pathogenesis” [3]. This assumption, that the scramblase activity merely helped to identify the ion channel as a potential therapeutic target, reflects a common opinion that syncytia produced in the body by infectious viruses are inconsequential.

To evaluate this assumption let us consider how cell fusion and syncytia it produces might be involved in COVID-19.

### Cell fusion as a trigger of the blood coagulation cascade

Discovering syncytia in COVID-19 patients led to a suggestion that “the fusogenic properties of the MERS-CoV- and SARS-CoV-2-infected cells might be linked to the pathogenesis of thrombosis,” [2] a major complication of COVID-19 [7, 8].

What could this link be?

I would like to suggest two candidates: the scramblase activity associated with spike-induced cell fusion, [3] and cell death.

Several observations suggest that the scramblase activity induced by spike [3] may be able to cause thrombosis.

**Figure 1:**
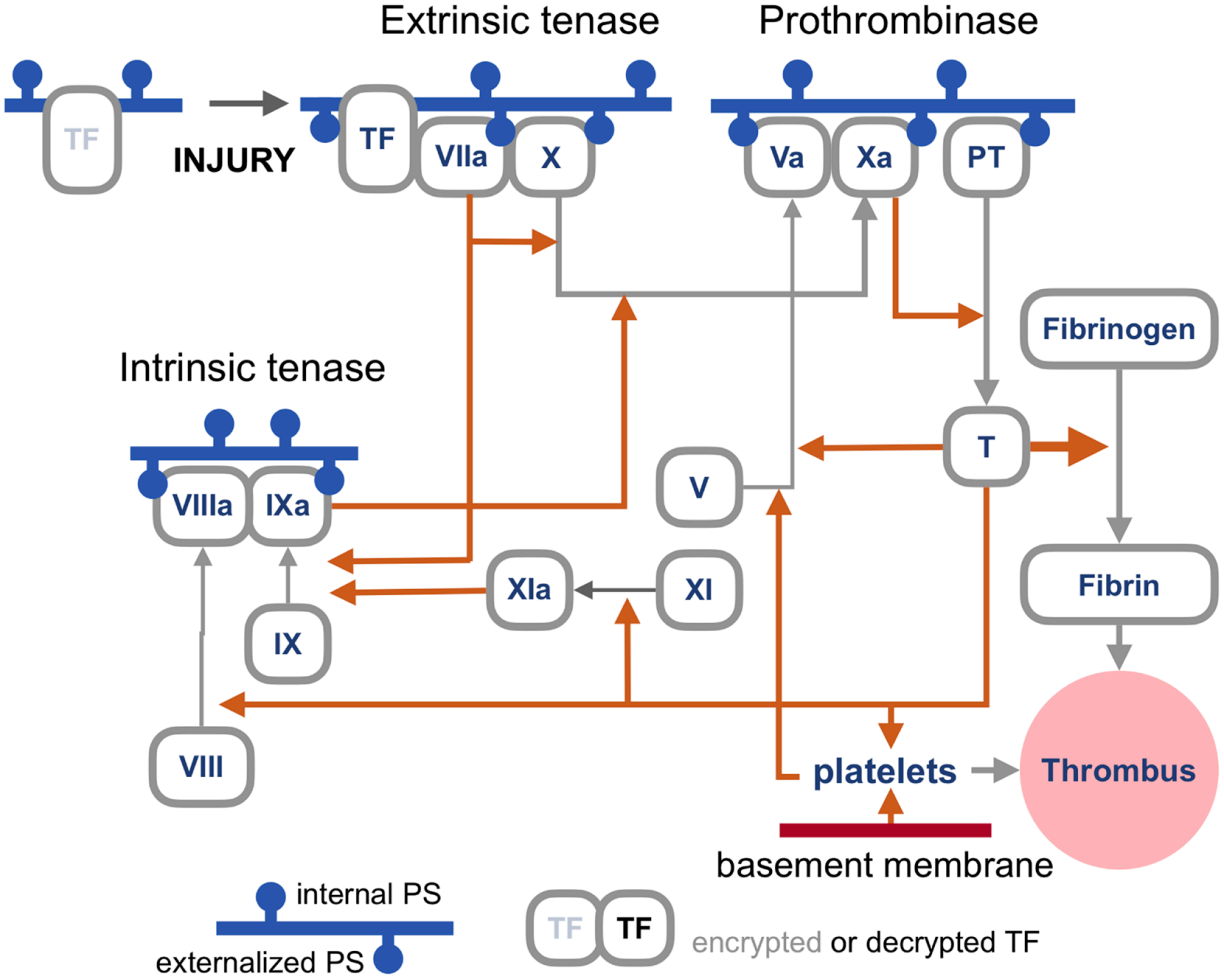
An outline of the blood coagulation cascade. Blood coagulation cascade is a network of proteases, their precursors, cofactors, cells, enzymes, feedbacks, and feedforwards whose complexity and still unresolved questions make this outline by necessity rudimentary, with the primary goal to illustrate where the proteins that require binding to externalized PS (phosphatidylserine) for activation are in the network. Most proteins involved in coagulation are called factors and are labeled by Roman numerals, such as Factor X or FX (hence enzymes that process FX are *tenases*). For simplicity, in this cartoon the letter F is omitted. Activated factors are labeled with an a, as in FXa. Orange arrows represent proteolytic activity, grey arrows show a transition between forms. Blue horizontal lines represent a cellular membrane with the cell surface facing down. Accordingly, the pinheads of externalized PS also face down. Note that most PS is actively relocated to face the cytoplasm unless the cell dies or the distribution is randomized by lipid scramblases. As discussed in the text, the primary trigger of coagulation induced by viral infections is the extrinsic tenase (top left), which is a complex of TF (Tissue Factor) and FVIIa assembled on externalized PS in the presence of calcium ions. This tenase produces FXa to activate enough thrombin to generate the components of the intrinsic tenase, which increases the production of FXa, and, consequently, of thrombin, which generates enough fibrin to make a thrombus, a meshwork of polymerized and cross-linked fibrin with entrapped blood cells, primarily platelets, which is large and stiff enough to obstruct a blood vessel. Note that TF is encrypted and so is unable to activate FVIIa, until it is de-encrypted by externalized PS [10].

First, PS externalization caused by scramblases not only enables cell fusion but also controls the rate limiting steps of the blood coagulation cascade [9–11] (Figure 1).

**Table:** 

Terminology and abbreviations	
***Cell fusion***	A process of merging two or more cells into one by merging their plasma membranes.
***Fusogen***	An agent, often a protein such as SARS-CoV-2 spike, capable of fusing cellular membranes. Viral fusogens fuse the viral envelope to the plasma membrane of the target cell and can fuse plasma membranes of adjacent cells to each other.
***Syncytium* ( **plural ***syncytia*) **	A multinucleated cell produced by the fusion of two or more cells. The term comes from Greek *syn* “together” and *kytos* “box, or cell”.
***Heterokaryon***	A syncytium produced from more than one cell type, say, a pneumocyte fused to an epithelial progenitor or a leukocyte.
***Homokaryon***	A syncytium produced from cells of the same type, as would be the case with the fusion of two or more pneumocytes.
***Cell hybrid***	Mononuclear offspring of syncytia, produced once a syncytium undergoes mitosis. For example, hybridomas are made by fusing leukocytes with plasmacytoma cells to obtain hybrids that produce monoclonal antibodies.
***PS (phosphatidylserine)***	The most abundant anionic (negatively charged) membrane lipid. In live cells, PS is actively moved to the cytoplasmic side of plasma membrane.
***Scramblases***	Proteins, such as TMEM16F, that randomize, or scramble, the asymmetric distribution of PS across the membrane, a process known as ***PS externalization***.

Second, a deficiency of TMEM16F, the scramblase identified by Braga and colleagues, is responsible for Scott syndrome, a bleeding disorder, [12] suggesting that this scramblase is involved in blood clotting.

Third, viral infections cause thrombosis primarily by triggering the assembly of what is called the fuse that triggers blood coagulation cascade [13, 14]. This fuse, also known as extrinsic tenase, is formed by Tissue Factor (TF) and Factor VIIa on the outer surface of the cellular membrane enriched in externalized PS [11] (Figure 1). The combination of PS, TF, and calcium ions can increase Factor VIIa activity by a remarkable five to eight orders of magnitude [15, 16].

Fourth, TF and its regulation have been considered potential targets for COVID-19 therapy [17, 18]. TF is regulated by controlling its expression, by tissue factor pathway inhibitor (TFPI), and by priming TF through a process known as de-encryption. The primary candidate for the de-encrypter is externalized PS [10]. How PS externalization is induced in TF-expressing cells is unclear [11].

### The syncytial tenase hypothesis

**Figure 2:**
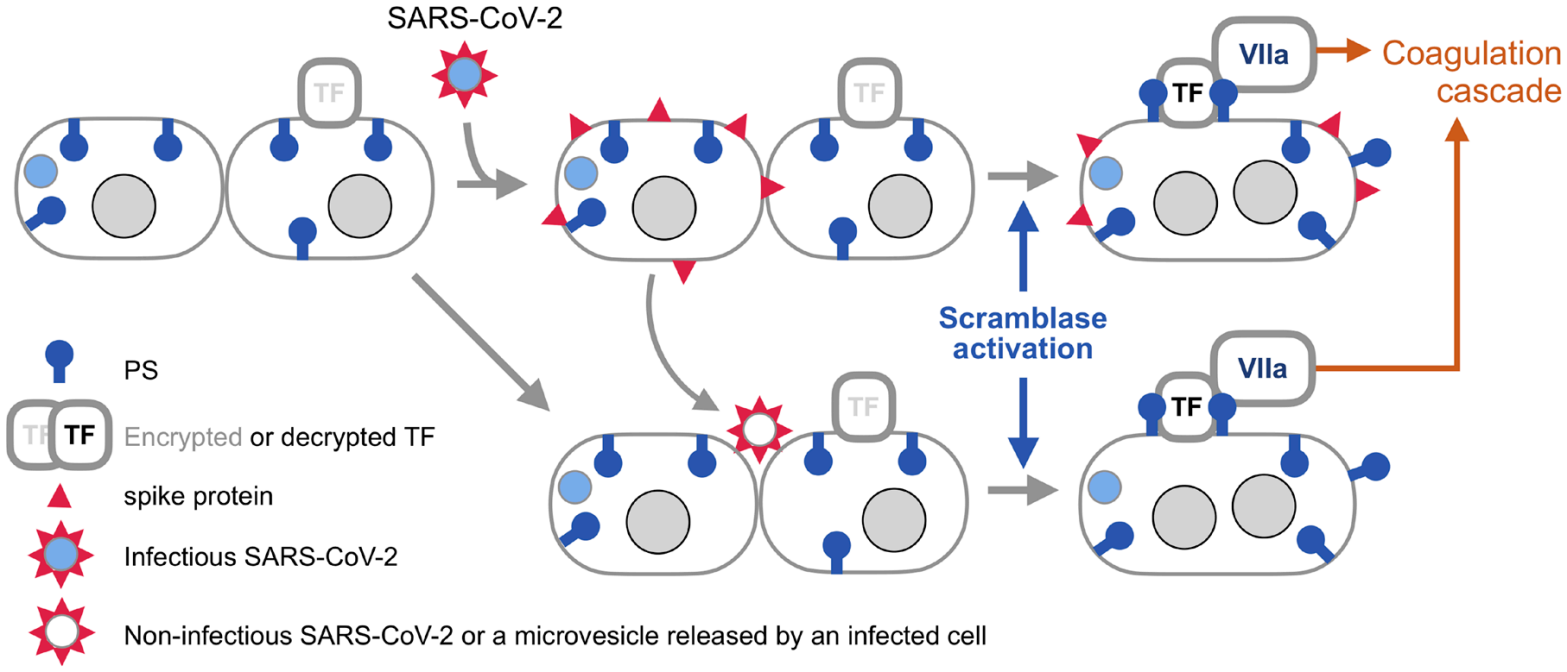
Syncytia induced by SARS-CoV-2 spike as a platform for triggering blood coagulation cascade. SARS-CoV-2 is covered by an envelope, which is fused to the cell membrane by spike once this protein is activated by binding to one of its receptors and processed by a membrane protease (both are not shown for simplicity). The infected cell produces viral components, including spike. Now, spike can fuse the membrane of the host cell with the membrane of an adjacent cell if that cell also has a spike receptor. Braga and colleagues [3] found that spike-induced cell fusion is associated with activation of TMEM16F, a scramblase that externalizes PS. This commentary proposes that PS externalized by spike enables the formation of the extrinsic tenase (Figure 1), the key trigger of blood coagulation cascade during viral infections. SARS-CoV-2 spike can also fuse cells if the virus is not infectious, or even if spike is incorporated into membrane vesicles, [22] like extracellular vesicles released by infected cells. This mechanism is known as fusion from without [21], as the viral particle or a vesicle provides a bridge between the membranes. Because syncytia produced by this mechanisms are not infected with SARS-CoV-2 in this case, their origin may be difficult to trace. Note that TF is encrypted, meaning that it is unable to activate FVIIa, until it is de-encrypted by externalized PS [10].

The synergy of TF and externalized PS in activating FVIIa, and the report that spike-induced syncytia externalize PS [3] together suggest a hypothesis that these syncytia can be a platform for assembling extrinsic tenase capable of triggering the blood coagulation cascade (Figure 2).

Three conditions would need to be met by this mechanism to cause clinically significant thrombosis. First, syncytia should express TF, which is likely because endothelial cells express TF during viral infections, [8] while the lungs are abundant in other cell types that express this protein [10]. Second, syncytia should come in contact with blood, which can happen if syncytia are formed within a blood vessel, or if blood vessels become leaky, a condition common in viral infections, including COVID-19 [19]. Finally, the tenase activity should be sufficiently abundant to trigger the coagulation cascade. What this tipping point is would depend on the number and size of syncytia, which will be defined by the extent of infection, as well as on the coagulation state of a patient.

### Thrombosis by death

Large syncytia made by viral fusogens are prone to die, at least in tissue culture. Hence, the second hypothesis suggests that syncytia formed by cells lining a blood vessel might contribute to thrombosis merely by dying because by sloughing off they would uncover a patch of the thrombogenic basement membrane with a surface area equal to that of many mononuclear cells. Because even a single 20-micron fiber of collagen, the main component of the basement membrane, is sufficient to trigger platelet-dependent clotting, [20] the patch exposed by dying syncytium that is made of more than several cells might be large enough to produce a thrombus by activating platelets (Figure 1, bottom right).

Given that even one thrombus can cause problems or even death, the potential contribution of syncytia to COVID-19 thrombosis by either activating the tenase or baring a patch of the basement membrane could be clinically relevant.

SARS-CoV-2 may be able to engage either mechanism both locally, by fusing infected cells, and remotely.

### Thrombosis at a distance

SARS-CoV-2 spike, like other viral fusogens, can fuse cells in two ways (Figure 2). To fuse from within, the virus makes an infected cell produce viral components, including spike, which is transported to the plasma membrane. Once there, spike can fuse the infected cell to an adjacent cell that has a spike receptor. Another mechanism, fusion from without, is executed by viral particles or lipid vesicles studded with spike that serve as a bridge between two cells [21, 22] This means that extracellular vesicles that are produced in COVID-19 patients [23] may be able to form syncytia and thus cause thrombosis even in tissues that are not infected with the virus.

### Inflammation and fibrosis

Besides mediating blood clotting, the coagulation cascade interrelates with signaling pathways that regulate inflammation, fibrosis, and some other conditions associated with COVID-19 [24]. Therefore, if syncytia produced by spike trigger the blood coagulation cascade, this activity would contribute to COVID-19 beyond inducing thrombosis.

### Cell fusion and SARS-CoV-2 variants of concern

While spike can fuse viruses to cells and cells to each other, the underlying mechanisms of these two activities are not identical. For example, sera from convalescent COVID-19 patients neutralize fusion of the virus to cells but fail to prevent the fusion of cells [22]. Likewise, modifying the spike of SARS-CoV, which causes severe acute respiratory syndrome, to enable maturation of this protein by furin, a protease that also processes SARS-CoV-2 spike, increases the ability of SARS-CoV to fuse cells manyfold with little effect on virus-cell fusion [25]. Finally, a single mutation in a porcine coronavirus spike enables this protein to cause cell-cell fusion at barely detectable amounts without affecting the ability of the virus to infect [26].

These observations mean that some SARS-CoV-2 variants can differ in the incidence of cell fusion and thus its consequences, including the ability to persist in the body by using cell-to-cell transmission, [27] a mechanism also used by HIV [28, 29]. This prediction is consistent with the report that novel SARS-CoV-2 variants of concern have mutations associated with a gain in syncytia formation [30].

### The pain of (con)fused neuronal networks

Neurological manifestations, including pain, are common in COVID-19 patients [31]. While SARS-CoV-2 is likely to contribute to these symptoms in multiple ways, short-circuiting neuronal networks by fusing neurons can explain not only how some neurological symptoms emerge but also why they last after the infection is cleared.

That fusing neurons can cause neurological problems has been considered in other virus-induced diseases. In animals, severe neurological symptoms of pseudorabies, a disease also known as mad itch, have been linked to the ability of pseudorabies virus to electrically couple the activity of neurons by fusing their axons [32, 33]. How such coupling could also contribute to the loss of smell, a common symptom of COVID-19, can be gleaned from experiments in the nematode *C. elegans* in which fusing two functionally different chemosensory neurons impaired chemosensation [34].

In humans, the fusion between neurons and glial cells, which surround neuronal bodies, has been proposed to explain the origin and persistence of the neuropathic pain that can last for months after the acute phase of herpes zoster (shingles) [35]. This fusion has been detected in a patient affected by shingles, [36] confirmed in a human xenograft model of this disease, [37] and accidentally discovered in an unrelated mouse model in which cortical neurons were infected with a retrovirus pseudotyped with VSV-G, the fusogen of vesicular stomatitis virus [38]. Whether the fusogens of human endogenous retroviruses (HERV), whose expression has been associated with various neurological disorders, function as pathogens of these diseases by fusing cells, as has been suggested, [39] is yet to be determined [40, 41]. Together, these observations mean that abnormal neuronal fusion induced by viral proteins is not restricted to a particular fusogen or to certain neurons.

Can SARS-CoV2 spike fuse neurons in the human body? Spike has been detected in the brain of deceased COVID-19 patients, [42] although how abundant SARS-CoV2 can be in the nervous system is still debated [43, 44]. However, considering how efficiently SARS-CoV2 spike fuses cells [22] and how intricate neuronal networks are, the chance that spike can disrupt them by fusing some of their components does not seem to be negligible, as a recent report also convincingly argues by demonstrating that spike can fuse neurons in brain organoids [45]. If spike retains this activity in the brain, it would be not difficult to envision how neuronal anastomoses created by cell fusion can contribute to cognitive disturbances associated with COVID-19 [31].

Such short-circuits may last for some time after a viral infection clears because the mechanisms that can repair them by “disconnecting” the anastomosed neurons or replacing them may be inefficient or inexistent.

### Syncytia and other products of cell fusion are heterogeneous abnormal cell types with emergent properties

Syncytia made by exogenous viruses are abnormal by definition because cell fusion in the body is normally restricted to a handful of physiological processes, such as fertilization, myogenesis, and the formation of osteoclasts, the cells that remodel bones [6, 46].

What is known about the mechanisms of physiological fusion – which is much less that one would expect given its function in the body – gives the impression that these mergers are planned and rehearsed down to the very last detail to ensure that only the right cells fuse at the right time and place and that, with the exception of fertilization and stem cell fusion, the resulting syncytia do not attempt to proliferate.

These sophisticated mechanisms, however, are overridden by many infectious viruses, including SARS CoV-2, which fuse cells randomly as long as the cells carry a cognate receptor [6, 47]. This randomness means that cell fusion induced by infectious viruses is a violent event that forcefully unites two or more finely tuned and specialized systems that just happened to be next to each other but may be quite different in their functions, gene expression patterns, cell cycle stage, age, activation status, and other features.

For example, in the lungs of COVID-19 patients, SARS-CoV-2 infects, and can thus fuse, multiple cell types: ciliated cells in the airway, alveolar type 2 pneumocytes, and epithelial progenitors among others [1]. What are the properties of, say, a syncytium that is made up of a pneumocyte with an epithelial progenitor? What happens if a leukocyte or another cell that carries one of spike receptors [48] joins in? Moreover, SARS-CoV-2 may not be the only virus that makes syncytia in COVID-19. For example, HERV-W ENV, the inactive fusogen of an endogenous retrovirus, [49] was detected in the leukocytes of COVID-19 patients at concentrations that exceeded that in the cells from healthy donors by orders of magnitude [50]. If SARS-CoV-2 also induces expression of related HERV ENVs, including fusogenic syncytin-1, [51] the number of cell types involved in cell fusion would increase even further. However, even without this potential boost, it is likely that COVID-19 patients have populations of diverse abnormal syncytia.

### What are the properties of abnormal syncytia?

The properties and the fates of syncytia produced by infectious viruses in the body remain practically unknown. However, observations made in experimental systems and by studying physiological syncytia provide some clues. One of them is that syncytia can become abnormal not only by combining distinct features of parental cells that are not found together in normal cell types, but also by having emergent properties that appear to result from reconciling distinct gene expression patterns underlying different cell types [52, 53].

For example, fusion of human bronchial epithelial cells to human multipotent stromal cells resulted in cells that appeared epithelial but failed to function properly because the two ion channels required to maintain bronchial and alveolar fluid balance were impaired due to changes in gene expression: one protein lacked a subunit, the other was improperly expressed [54]. Likewise, bone marrow-derived cells fused to hepatocytes in a mouse model of chronic liver damage yielded cells that differed in their gene expression patterns from both parental types and, unexpectedly, expressed cytokines and genes involved in neurotransmission and in the TGF-β pathway [55]. In an extreme case illustrating an enigmatic phenomenon called extinction, fusion of hepatoma cells to fibroblasts silenced hundreds of genes specific to either parental cell type, thus producing dedifferentiated cells [56]. Even fusion of cells belonging to the same cell type can produce syncytia with new properties, as happens with osteoclasts, which resorb bone better than their mononuclear precursors [57].

Given the outlined examples, it is not unreasonable to envision that some syncytia created by SARS CoV-2, associated viral infections, or induced endogenous viruses can produce cytokines or other signaling factors capable of deregulating tissue homeostasis either locally or even systemically, as happens in COVID-19 during cytokine storms [24]. These cells might also become sanctuaries for the virus, as has been reported for HIV [ 28,29], or by evading or corrupting immune surveillance, perhaps by fusing to immune cells.

### Cell fusion and neoplasia

While the published reports on COVID-19 discuss large syncytia, as these cells are the most noticeable products of cell fusion due to their size and numerous nuclei (thus often called multinucleated giant cells), they are not the only outcome of cell fusion. Cell fusion can produce binuclear or trinuclear cells, which are often more abundant in experimental systems than large syncytia but could go unnoticed in human tissues. Even if noticed, they may not be attributed to cell fusion because distinguishing them reliably from binuclear cells produced by failed mitosis in human tissues may be difficult or impossible with available tools [58].

A syncytium, especially if it has only two or three nuclei, can enter mitosis to produce mononuclear daughter cells. These mitoses are commonly multipolar and thus are prone to producing aneuploid cells with chromosomal aberrations, adding another layer of abnormal features to the offspring of cell fusion [59, 60]. Such abnormalities may be particularly significant to COVID-19 patients with neoplastic lesions because chromosomal aberrations contribute to tumor progression [61, 62], as do epigenetic abnormalities found in the products of cell fusion [53].

Another potential concern comes from a long-standing model that cell fusion, particularly fusion induced by viruses, contributes to cancer development, progression, metastasis, recurrence, dormancy, and acquired drug resistance (reviewed in: [39, 63–65]). This model has been supported by recent reports of cell hybrids in human cancers, [58, 66] by multiple observations in animal models (reviewed in: [39, 65, 67, 68]), by findings that human cells can be made cancerous through cell fusion, [59, 69, 70] and by comparing the evolution of tumors and cell hybrids [71]. However, whether any neoplastic hybrids found in humans [58] are made by viral fusogens, as has been suggested [72–74], is yet to be determined. Nonetheless, out of an abundance of caution, it may be reasonable to monitor the incidence and progression of neoplastic lesions in COVID-19 patients closely, as has been proposed [75].

This incomplete list of mechanisms that cell fusion can use to produce diverse abnormal cells, including neoplastic, suggests that drugs that target cell fusion, such as those identified by Braga and colleagues [3], might also be useful for preventing potential neoplastic consequences of COVID-19. These drugs, as we about to discuss, can also be useful for preventing side effects of some COVID-19 vaccines.

### Cell fusion and COVID-19 vaccines

The majority of available COVID-19 vaccines, including all four vaccines authorized in the United States and the European Union, work by expressing spike in the cells of the injected individuals. They do so either by infecting the cells with an adenovirus carrying a spike gene (AstraZeneca [76] and Janssen [77] vaccines) or by transfecting them with a spike mRNA (Pfizer[78] and Moderna [79] vaccines). Once expressed, spike is recognized by the immune system as a foreign antigen, triggering an immune response to the protein and thus to SARS-CoV-2.

Considering spike as an antigen might distract from the fact that the primary activity of this protein is to fuse biological membranes, which is why spike expressed in cells can fuse them into syncytia. This fact raises two questions that have yet to be asked despite all the attention and scrutiny that spike has received [80]. Does spike fuse any cells if expressed by the vaccines? And, does this fusion, should it occur, have any unwanted consequences?

Given that spike expressed by SARS-CoV-2 fuses cells in COVID-19 patients, [1, 2] that spike expressed by viral vectors or by transfection fuses human cells in the dish, [22, 81, 82] and that spike fuses cells even if expressed in undetectable amounts, [22] it is reasonable to presume, until proven otherwise, that spike does fuse some cells in the injected individuals.

### Could this fusion be pathogenic?

If cell fusion induced by expression of spike contributes to COVID-19 complications, as this and previous reports [1, 2] have suggested, then expressing spike by other means, including those used by the vaccines, should be expected to have similar effects. The puzzling case of the AstraZeneca vaccine is consistent with this possibility.

**Figure 3:**
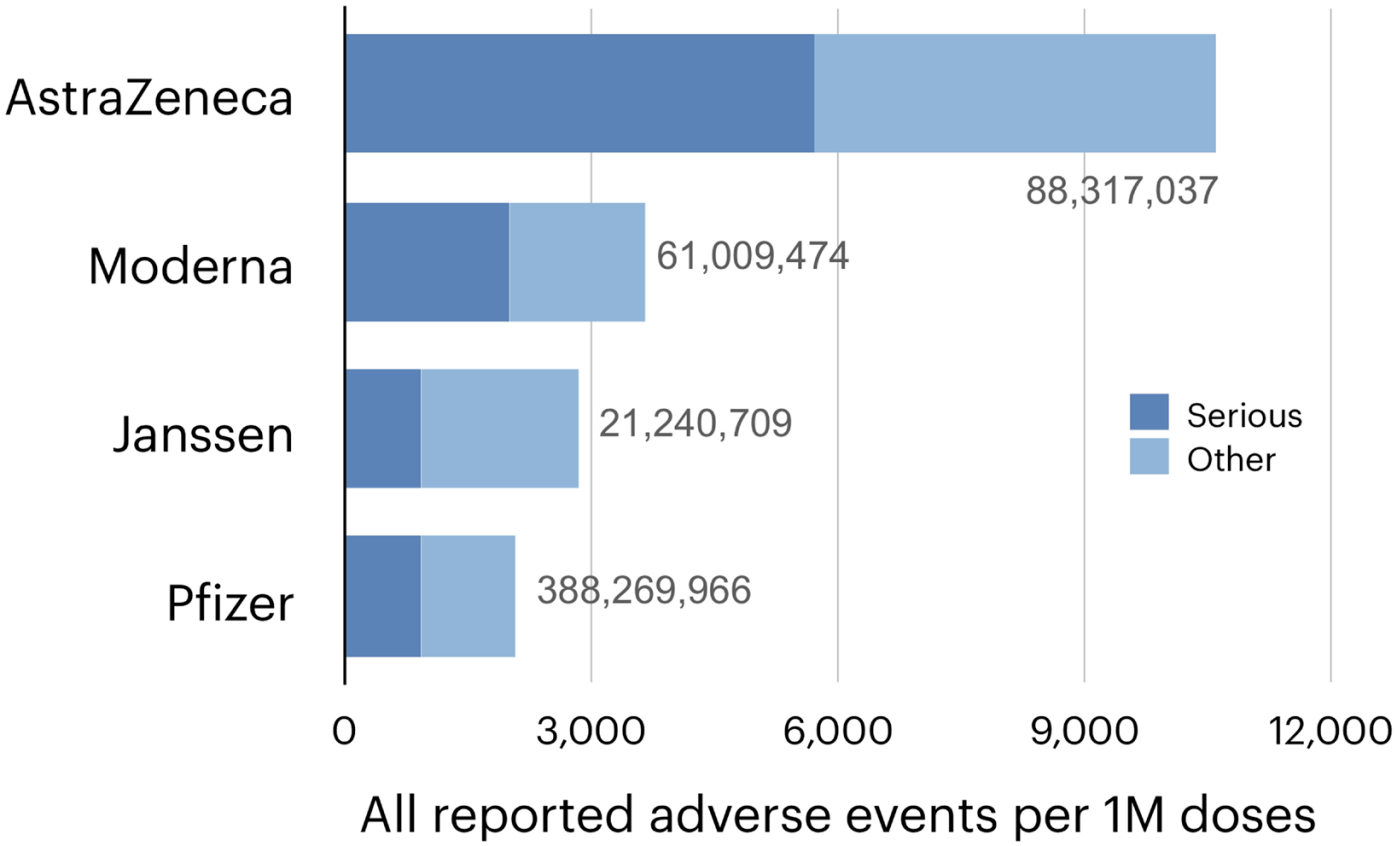
The incidence of suspected vaccine complications recorded in the European database of suspected adverse drug reactions reports (EudraVigilance) [109] as of August 6, 2021. The numbers of doses administered by that date, and shown next to the bars. were taken from: https://vaccinetracker.ecdc.europa.eu/public/extensions/COVID-19/vaccine-tracker.html#distribution-tab.

An unexplained feature of this vaccine is the highest incidence of reported complications among the four vaccines [83, 84] (Figure 3), including a series of thrombotic complications [85, 86] that permanently suspended the AstraZeneca vaccine in a number of countries and has delayed its authorization in the United States [87]. These complications have been ascribed to antibodies elicited by adenoviruses against platelet factor 4, [88, 89] to the alternative splicing of spike, [90] and to the binding of adenoviruses or spike to platelets [80, 89]. However, the proposed mechanisms still need to fully explain why thrombotic events have also been reported for mRNA vaccines, albeit at a lower incidence, [83, 91] why they can occur within days after vaccination, [83] why they are as rare as they fortunately are, why the AstraZeneca vaccine has a higher incidence not only of thrombosis but also of some other complications [83], and, finally, how these complications can be prevented.

If spike-induced cell fusion is pathogenic, as this commentary argues, then the unfortunate ranking of the AstraZeneca vaccine becomes predictable because it is the only out of the four vaccines that makes the wild type, fully fusogenic spike, [76, 92] delivering it with a vector optimized to express “very high levels” of the protein [93].

Spike used in the other three vaccines has been made less fusogenic, apparently fortuitously, while optimizing spike as an antigen. To improve the immune response, the developers [76, 77, 79] have stabilized spike structure by two mutations that suppress a conformational change triggered by binding to ACE2 [94, 95]. Because this change is involved in spike activation, [48] suppressing it has also inhibited cell fusion in a tissue culture assay by several fold [77]. Two additional mutations introduced in the Janssen vaccine decreased this incidence in the same assay further [77] by altering the site recognized by furin, a protease that contributes to spike activation by cleaving it into two subunits [48]. Whether these additional mutations remain as effective in the human body is unclear, as other proteases can replace furin [96, 97] and because this cleavage may be not required [98]. However, since the abundance of these proteases varies among human tissues, [97] altering the furin site might affect the incidence or location of some complications. None of the developers mutated the S2’ site, whose cleavage exposes the fusion peptide, the part of viral fusogens that penetrates the target membrane [99].

These observations suggest a relationship between the fusogenicity of spike proteins and the reported incidence of side effects. Is this relationship accidental or causal?

A causal relationship entails two testable predictions:

First, complementing vaccination with drugs that prevent spike-induced cell fusion should reduce the incidence of complications, particularly for the AstraZeneca vaccine and other vaccines that express wild type spike [100]. A number of approved drugs that have such activity, including hotly debated ivermectin, [101–102] have been already identified by Braga and colleagues [3].

Second, vaccines that use recombinant spike fragments, [103] or other derivatives of spike that are not fusogenic without any doubt, should have fewer complications than vaccines that express fully or partially fusogenic spike. Vaccines that use inactivated SARS-CoV2 (currently Sinopharm [104] and Sinovac [105]) would have an intermediate incidence of complications because inactivated viruses can still fuse cells from without (Figure 2), although without the capacity to express spike the incidence of syncytia is limited by the number of injected viral particles. This prediction is consistent with safety reports for vaccines that use inactivated virus [106, 107] but further studies, and having all current and future vaccines tracked by publicly accessible databases of suspected vaccine complications, like VAERS [108] in the United States and EudraVigilance [109] in Europe, would provide a larger set of data to evaluate, as has been done to compare the AstraZeneca and Pfizer vaccines [83].

The hypothesis that cell fusion mediates some vaccine complications needs to be tested because the scale of COVID-19 vaccination calls for an abundance of caution, which hardly implies overlooking the primary activity of the antigen, and because the proposed hypothesis applies to other fusogenic proteins that one might want to express in the human body to prevent a viral infection or another disease. For example, a number of such vaccines are already in Moderna’s pipeline [110]. Evaluating the potential consequences of cell fusion early in vaccine development might help to prevent avoidable side effects.

If vaccines cause vaccine complications by inducing cell fusion, one might ask, why then are these complications so rare, diverse, and overlap with those observed in COVID-19? Perhaps, as has been suggested, [111] the outcome of vaccination depends on how a vaccine is injected. If, as intended, the vaccine stays strictly intramuscular, the syncytia it creates may be inconsequential as they stay at the site of injection and die in due course. However, if a vaccine spreads systemically because it is accidentally injected into a blood or lymphatic vessel, or for other reasons, the outcome would depend on which cells, where, and in what numbers begin to express spike and thus acquire the ability to fuse. For example, the fusion of endothelial cells to each other or to other cells carrying a spike receptor, including platelets [112] and pericytes [113], could result in thrombosis, while the fusion of neurons may lead to neurological manifestations. Some complications caused by cell fusion may be specific to a particular vaccine or to COVID-19 because the tropisms of adenoviral vectors, mRNA-carrying lipid particles, and SARS-CoV-2 overlap but are not identical [48, 114, 115] A contributing factor could be a predisposition of some individuals to cell fusion by viral fusogens, which is difficult to evaluate at this time because cell fusion regulation in general and the regulation of cell fusion induced by viruses in particular is still largely a *terra incognita*.

In summary, this author hopes that the discovery of syncytia in COVID-19 patients will help to dissect cell fusion and its consequences, both in health and in disease, by making more researchers aware of this fascinating yet often overlooked process. After all, we tend to notice only what we expect to see.

## References

[1] Buchrieser J , Dufloo J , Hubert M , Monel B , Planas D , Rajah MM , Planchais C , Porrot F , Guivel-Benhassine F , Van der Werf S , Casartelli N , Mouquet H , Bruel T , Schwartz O . Syncytia formation by SARS-CoV-2-infected cells. EMBO J. 2020; 39:e106267. 10.15252/embj.2020106267. 33051876 PMC7646020

[2] Bussani R , Schneider E , Zentilin L , Collesi C , Ali H , Braga L , Volpe MC , Colliva A , Zanconati F , Berlot G , Silvestri F , Zacchigna S , Giacca M . Persistence of viral RNA, pneumocyte syncytia and thrombosis are hallmarks of advanced COVID-19 pathology. EBioMedicine. 2020; 61:103104. 10.1016/j.ebiom.2020.103104. 33158808 PMC7677597

[3] Braga L , Ali H , Secco I , Chiavacci E , Neves G , Goldhill D , Penn R , Jimenez-Guardeño JM , Ortega-Prieto AM , Bussani R , Cannatà A , Rizzari G , Collesi C , et al. Drugs that inhibit TMEM16 proteins block SARS-CoV-2 spike-induced syncytia. Nature. 2021; 594:88–93. 10.1038/s41586-021-03491-6. 33827113 PMC7611055

[4] Le T , Jia Z , Le SC , Zhang Y , Chen J , Yang H . An inner activation gate controls TMEM16F phospholipid scrambling. Nat Commun. 2019; 10:1846. 10.1038/s41467-019-09778-7. 31015464 PMC6478717

[5] Zhang Y , Le T , Grabau R , Mohseni Z , Kim H , Natale DR , Feng L , Pan H , Yang H . TMEM16F phospholipid scramblase mediates trophoblast fusion and placental development. Sci Adv. 2020; 6:eaba0310. 10.1126/sciadv.aba0310. 32494719 PMC7202889

[6] Brukman NG , Uygur B , Podbilewicz B , Chernomordik LV . How cells fuse. J Cell Biol. 2019; 218:1436–51. 10.1083/jcb.201901017. 30936162 PMC6504885

[7] Hanff TC , Mohareb AM , Giri J , Cohen JB , Chirinos JA . Thrombosis in COVID-19. Am J Hematol. 2020; 95:1578–89. 10.1002/ajh.25982. 32857878 PMC7674272

[8] Mackman N , Antoniak S , Wolberg AS , Kasthuri R , Key NS . Coagulation Abnormalities and Thrombosis in Patients Infected With SARS-CoV-2 and Other Pandemic Viruses. Arterioscler Thromb Vasc Biol. 2020; 40:2033–44. 10.1161/ATVBAHA.120.314514. 32657623 PMC7447001

[9] Zwaal RF , Comfurius P , Bevers EM . Lipid-protein interactions in blood coagulation. Biochim Biophys Acta. 1998; 1376:433–53. 10.1016/s0304-4157(98)00018-5. 9805008

[10] Grover SP , Mackman N . Tissue Factor: An Essential Mediator of Hemostasis and Trigger of Thrombosis. Arterioscler Thromb Vasc Biol. 2018; 38:709–25. 10.1161/ATVBAHA.117.309846. 29437578

[11] Smith SA , Morrissey JH . Interactions Between Platelets and the Coagulation System. In: Michelson AD , editor. Platelets (Fourth Edition). Academic Press; 2019; 393–400. 10.1016/B978-0-12-813456-6.00021-7.

[12] Suzuki J , Umeda M , Sims PJ , Nagata S . Calcium-dependent phospholipid scrambling by TMEM16F. Nature. 2010; 468:834–38. 10.1038/nature09583. 21107324

[13] Furie B , Furie BC . Mechanisms of thrombus formation. N Engl J Med. 2008; 359:938–49. 10.1056/NEJMra0801082. 18753650

[14] Antoniak S , Mackman N . Multiple roles of the coagulation protease cascade during virus infection. Blood. 2014; 123:2605–13. 10.1182/blood-2013-09-526277. 24632711 PMC3999750

[15] Bom VJ , Bertina RM . The contributions of Ca2+, phospholipids and tissue-factor apoprotein to the activation of human blood-coagulation factor X by activated factor VII. Biochem J. 1990; 265:327–36. 10.1042/bj2650327. 2302175 PMC1136891

[16] Smith SA , Travers RJ , Morrissey JH . How it all starts: Initiation of the clotting cascade. Crit Rev Biochem Mol Biol. 2015; 50:326–36. 10.3109/10409238.2015.1050550. 26018600 PMC4826570

[17] DiNicolantonio JJ , McCarty M . Thrombotic complications of COVID-19 may reflect an upregulation of endothelial tissue factor expression that is contingent on activation of endosomal NADPH oxidase. Open Heart. 2020; 7:e001337. 10.1136/openhrt-2020-001337. 32532805 PMC7298678

[18] Cañas CA , Cañas F , Bautista-Vargas M , Bonilla-Abadía F . Role of Tissue Factor in the Pathogenesis of COVID-19 and the Possible Ways to Inhibit It. Clin Appl Thromb Hemost. 2021; 27:10760296211003983. 10.1177/10760296211003983. 33784877 PMC8020089

[19] Butsabong T , Felippe M , Campagnolo P , Maringer K . The emerging role of perivascular cells (pericytes) in viral pathogenesis. J Gen Virol. 2021; 102. 10.1099/jgv.0.001634. 34424156 PMC8513640

[20] Zhu S , Tomaiuolo M , Diamond SL . Minimum wound size for clotting: flowing blood coagulates on a single collagen fiber presenting tissue factor and von Willebrand factor. Integr Biol (Camb). 2016; 8:813–20. 10.1039/c6ib00077k. 27339024 PMC4980166

[21] Gallaher WR , Bratt MA . Temperature-dependent inhibition of fusion from without. J Virol. 1972; 10:159–61. 10.1128/JVI.10.1.159-161.1972. 5064776 PMC356440

[22] Theuerkauf SA , Michels A , Riechert V , Maier TJ , Flory E , Cichutek K , Buchholz CJ . Quantitative assays reveal cell fusion at minimal levels of SARS-CoV-2 spike protein and fusion from without. iScience. 2021; 24:102170. 10.1016/j.isci.2021.102170. 33585805 PMC7871100

[23] Rosell A , Havervall S , von Meijenfeldt F , Hisada Y , Aguilera K , Grover SP , Lisman T , Mackman N , Thålin C . Patients With COVID-19 Have Elevated Levels of Circulating Extracellular Vesicle Tissue Factor Activity That Is Associated With Severity and Mortality-Brief Report. Arterioscler Thromb Vasc Biol. 2021; 41:878–82. 10.1161/atvbaha.120.315547. 33267656 PMC7837685

[24] Sriram K , Insel PA . Inflammation and thrombosis in COVID-19 pathophysiology: proteinase-activated and purinergic receptors as drivers and candidate therapeutic targets. Physiol Rev. 2021; 101:545–67. 10.1152/physrev.00035.2020. 33124941 PMC8238137

[25] Follis KE , York J , Nunberg JH . Furin cleavage of the SARS coronavirus spike glycoprotein enhances cell-cell fusion but does not affect virion entry. Virology. 2006; 350:358–69. 10.1016/j.virol.2006.02.003. 16519916 PMC7111780

[26] Wanitchang A , Saenboonrueng J , Kaewborisuth C , Srisutthisamphan K , Jongkaewwattana A . A Single V672F Substitution in the Spike Protein of Field-Isolated PEDV Promotes Cell-Cell Fusion and Replication in VeroE6 Cells. Viruses. 2019; 11:282. 10.3390/v11030282. 30897856 PMC6466060

[27] Zeng C , Evans JP , King T , Zheng YM , Oltz EM , Whelan SPJ , Saif L , Peeples ME , Liu SL . SARS-CoV-2 Spreads through Cell-to-Cell Transmission. bioRxiv. 2021; 2021.06.01.446579. 10.1101/2021.06.01.446579. 34937699 PMC8740724

[28] Symeonides M , Murooka TT , Bellfy LN , Roy NH , Mempel TR , Thali M . HIV-1-Induced Small T Cell Syncytia Can Transfer Virus Particles to Target Cells through Transient Contacts. Viruses. 2015; 7:6590–603. 10.3390/v7122959. 26703714 PMC4690882

[29] Bracq L , Xie M , Benichou S , Bouchet J . Mechanisms for Cell-to-Cell Transmission of HIV-1. Front Immunol. 2018; 9:260. 10.3389/fimmu.2018.00260. 29515578 PMC5825902

[30] Escalera A , Gonzalez-Reiche AS , Aslam S , Mena I , Pearl RL , Laporte M , Fossati A , Rathnasinghe R , Alshammary H , van de Guchte A , Bouhaddou M , Kehrer T , Zuliani-Alvarez L , et al. SARS-CoV-2 variants of concern have acquired mutations associated with an increased spike cleavage. Microbiology. 2021. http://biorxiv.org/lookup/doi/10.1101/2021.08.05.455290. 10.1016/j.chom.2022.01.006 PMC8776496 35150638

[31] Solomon T . Neurological infection with SARS-CoV-2 - the story so far. Nat Rev Neurol. 2021; 17:65–66. 10.1038/s41582-020-00453-w. 33414554 PMC7789883

[32] Granstedt AE , Bosse JB , Thiberge SY , Enquist LW . In vivo imaging of alphaherpesvirus infection reveals synchronized activity dependent on axonal sorting of viral proteins. Proc Natl Acad Sci U S A. 2013; 110:E3516–25. 10.1073/pnas.1311062110. 23980169 PMC3773797

[33] McCarthy KM , Tank DW , Enquist LW . Pseudorabies virus infection alters neuronal activity and connectivity *in vitro* . PLoS Pathog. 2009; 5:e1000640. 10.1371/journal.ppat.1000640. 19876391 PMC2763221

[34] Giordano-Santini R , Kaulich E , Galbraith KM , Ritchie FK , Wang W , Li Z , Hilliard MA . Fusogen-mediated neuron-neuron fusion disrupts neural circuit connectivity and alters animal behavior. Proc Natl Acad Sci U S A. 2020; 117:23054–65. 10.1073/pnas.1919063117. 32855296 PMC7502713

[35] Zerboni L , Sen N , Oliver SL , Arvin AM . Molecular mechanisms of varicella zoster virus pathogenesis. Nat Rev Microbiol. 2014; 12:197–210. 10.1038/nrmicro3215. 24509782 PMC4066823

[36] Esiri MM , Tomlinson AH . Herpes Zoster. Demonstration of virus in trigeminal nerve and ganglion by immunofluorescence and electron microscopy. J Neurol Sci. 1972; 15:35–48. 10.1016/0022-510x(72)90120-7. 4332851

[37] Reichelt M , Zerboni L , Arvin AM . Mechanisms of varicella-zoster virus neuropathogenesis in human dorsal root ganglia. J Virol. 2008; 82:3971–83. 10.1128/JVI.02592-07. 18256143 PMC2292995

[38] Ackman JB , Siddiqi F , Walikonis RS , LoTurco JJ . Fusion of microglia with pyramidal neurons after retroviral infection. J Neurosci. 2006; 26:11413–22. 10.1523/JNEUROSCI.3340-06.2006. 17079670 PMC6674527

[39] Duelli D , Lazebnik Y . Cell fusion: a hidden enemy? Cancer Cell. 2003; 3:445–48. 10.1016/s1535-6108(03)00114-4. 12781362

[40] Geis FK , Goff SP . Silencing and Transcriptional Regulation of Endogenous Retroviruses: An Overview. Viruses. 2020; 12:884. 10.3390/v12080884. 32823517 PMC7472088

[41] Giménez-Orenga K , Oltra E . Human Endogenous Retrovirus as Therapeutic Targets in Neurologic Disease. Pharmaceuticals (Basel). 2021; 14:495. 10.3390/ph14060495. 34073730 PMC8225122

[42] Song E , Zhang C , Israelow B , Lu-Culligan A , Prado AV , Skriabine S , Lu P , Weizman OE , Liu F , Dai Y , Szigeti-Buck K , Yasumoto Y , Wang G , et al. Neuroinvasion of SARS-CoV-2 in human and mouse brain. J Exp Med. 2021; 218:e20202135. 10.1084/jem.20202135. 33433624 PMC7808299

[43] Puelles VG , Lütgehetmann M , Lindenmeyer MT , Sperhake JP , Wong MN , Allweiss L , Chilla S , Heinemann A , Wanner N , Liu S , Braun F , Lu S , Pfefferle S , et al. Multiorgan and Renal Tropism of SARS-CoV-2. N Engl J Med. 2020; 383:590–92. 10.1056/NEJMc2011400. 32402155 PMC7240771

[44] Thakur KT , Miller EH , Glendinning MD , Al-Dalahmah O , Banu MA , Boehme AK , Boubour AL , Bruce SS , Chong AM , Claassen J , Faust PL , Hargus G , Hickman RA , et al. COVID-19 neuropathology at Columbia University Irving Medical Center/New York Presbyterian Hospital. Brain. 2021 Apr 15. 10.1093/brain/awab148. [Epub ahead of print]. 33856027 PMC8083258

[45] Martinez-Marmol R , Giordano-Santini R , Kaulich E , Cho AN , Riyadh MA , Robinson E , Balistreri G , Meunier FA , Ke YD , Ittner LM , Hilliard MA . The SARS-CoV-2 spike (S) and the orthoreovirus p15 cause neuronal and glial fusion. bioRxiv. 2021; 2021.09.01.458544. https://www.biorxiv.org/content/10.1101/2021.09.01.458544v1.

[46] Petrany MJ , Millay DP . Cell Fusion: Merging Membranes and Making Muscle. Trends Cell Biol. 2019; 29:964–73. 10.1016/j.tcb.2019.09.002. 31648852 PMC7849503

[47] Hernández JM , Podbilewicz B . The hallmarks of cell-cell fusion. Development. 2017; 144:4481–95. 10.1242/dev.155523. 29254991

[48] Peng R , Wu LA , Wang Q , Qi J , Gao GF . Cell entry by SARS-CoV-2. Trends Biochem Sci. 2021; 46:848–60. 10.1016/j.tibs.2021.06.001. 34187722 PMC8180548

[49] Charvet B , Pierquin J , Brunel J , Gorter R , Quétard C , Horvat B , Amor S , Portoukalian J , Perron H . Human Endogenous Retrovirus Type W Envelope from Multiple Sclerosis Demyelinating Lesions Shows Unique Solubility and Antigenic Characteristics. Virol Sin. 2021 Mar 26. 10.1007/s12250-021-00372-0. [Epub ahead of print]. 33770381 PMC8558138

[50] Balestrieri E , Minutolo A , Petrone V , Fanelli M , Iannetta M , Malagnino V , Zordan M , Vitale P , Charvet B , Horvat B , Bernardini S , Garaci E , di Francesco P , et al. Evidence of the pathogenic HERV-W envelope expression in T lymphocytes in association with the respiratory outcome of COVID-19 pa-tients. EBioMedicine. 2021; 66:103341. 10.1016/j.ebiom.2021.103341. 33867312 PMC8082064

[51] Blond JL , Lavillette D , Cheynet V , Bouton O , Oriol G , Chapel-Fernandes S , Mandrand B , Mallet F , Cosset FL . An envelope glycoprotein of the human endogenous retrovirus HERV-W is expressed in the human placenta and fuses cells expressing the type D mammalian retrovirus receptor. J Virol. 2000; 74:3321–29. 10.1128/jvi.74.7.3321-3329.2000. 10708449 PMC111833

[52] Koulakov AA , Lazebnik Y . The problem of colliding networks and its relation to cell fusion and cancer. Biophys J. 2012; 103:2011–20. 10.1016/j.bpj.2012.08.062. 23199929 PMC3491708

[53] Lazebnik Y . The shock of being united and symphiliosis. Another lesson from plants? Cell Cycle. 2014; 13:2323–29. 10.4161/cc.29704. 25483182 PMC4128876

[54] Curril IM , Koide M , Yang CH , Segal A , Wellman GC , Spees JL . Incomplete reprogramming after fusion of human multipotent stromal cells and bronchial epithelial cells. FASEB J. 2010; 24:4856–64. 10.1096/fj.09-152991. 20724526 PMC2992376

[55] Quintana-Bustamante O , Grueso E , Garcia-Escudero R , Arza E , Alvarez-Barrientos A , Fabregat I , Garcia-Bravo M , Meza NW , Segovia JC . Cell fusion reprogramming leads to a specific hepatic expression pattern during mouse bone marrow derived hepatocyte formation *in vivo* . PLoS One. 2012; 7:e33945. 10.1371/journal.pone.0033945. 22457803 PMC3311566

[56] Bulla GA , Luong Q , Shrestha S , Reeb S , Hickman S . Genome-wide analysis of hepatic gene silencing in mammalian cell hybrids. Genomics. 2010; 96:323–32. 10.1016/j.ygeno.2010.08.006. 20801210

[57] Yagi M , Miyamoto T , Sawatani Y , Iwamoto K , Hosogane N , Fujita N , Morita K , Ninomiya K , Suzuki T , Miyamoto K , Oike Y , Takeya M , Toyama Y , Suda T . DC-STAMP is essential for cell-cell fusion in osteoclasts and foreign body giant cells. J Exp Med. 2005; 202:345–51. 10.1084/jem.20050645. 16061724 PMC2213087

[58] Gast CE , Silk AD , Zarour L , Riegler L , Burkhart JG , Gustafson KT , Parappilly MS , Roh-Johnson M , Goodman JR , Olson B , Schmidt M , Swain JR , Davies PS , et al. Cell fusion potentiates tumor heterogeneity and reveals circulating hybrid cells that correlate with stage and survival. Sci Adv. 2018; 4:eaat7828. 10.1126/sciadv.aat7828. 30214939 PMC6135550

[59] Duelli DM , Padilla-Nash HM , Berman D , Murphy KM , Ried T , Lazebnik Y . A virus causes cancer by inducing massive chromosomal instability through cell fusion. Curr Biol. 2007; 17:431–37. 10.1016/j.cub.2007.01.049. 17320392

[60] Godinho SA , Kwon M , Pellman D . Centrosomes and cancer: how cancer cells divide with too many centrosomes. Cancer Metastasis Rev. 2009; 28:85–98. 10.1007/s10555-008-9163-6. 19156503

[61] Gemoll T , Auer G , Ried T , Habermann JK . Genetic Instability and Disease Prognostication. Recent Results Cancer Res. 2015; 200:81–94. 10.1007/978-3-319-20291-4_4. 26376873 PMC7737009

[62] Baudoin NC , Bloomfield M . Karyotype Aberrations in Action: The Evolution of Cancer Genomes and the Tumor Microenvironment. Genes (Basel). 2021; 12:558. 10.3390/genes12040558. 33921421 PMC8068843

[63] Shabo I , Svanvik J , Lindström A , Lechertier T , Trabulo S , Hulit J , Sparey T , Pawelek J . Roles of cell fusion, hybridization and polyploid cell formation in cancer metastasis. World J Clin Oncol. 2020; 11:121–35. 10.5306/wjco.v11.i3.121. 32257843 PMC7103524

[64] Parris GE . Historical perspective of cell-cell fusion in cancer initiation and progression. Crit Rev Oncog. 2013; 18:1–18. 10.1615/critrevoncog.v18.i1-2.20. 23237550

[65] Sieler M , Weiler J , Dittmar T . Cell-Cell Fusion and the Roads to Novel Properties of Tumor Hybrid Cells. Cells. 2021; 10:1465. 10.3390/cells10061465. 34207991 PMC8230653

[66] Laberge GS , Duvall E , Haedicke K , Pawelek J . Leukocyte-Cancer Cell Fusion-Genesis of a Deadly Journey. Cells. 2019; 8:170. 10.3390/cells8020170. 30781683 PMC6406780

[67] Pawelek JM , Chakraborty AK . Fusion of tumour cells with bone marrow-derived cells: a unifying explanation for metastasis. Nat Rev Cancer. 2008; 8:377–86. 10.1038/nrc2371. 18385683

[68] Noubissi FK , Ogle BM . Cancer Cell Fusion: Mechanisms Slowly Unravel. Int J Mol Sci. 2016; 17:1587. 10.3390/ijms17091587. 27657058 PMC5037852

[69] Delespaul L , Merle C , Lesluyes T , Lagarde P , Le Guellec S , Pérot G , Baud J , Carlotti M , Danet C , Fèvre M , Rousseau B , Durrieu S , Teichmann M , et al. Fusion-mediated chromosomal instability promotes aneuploidy patterns that resemble human tumors. Oncogene. 2019; 38:6083–94. 10.1038/s41388-019-0859-6. 31270395

[70] Lartigue L , Merle C , Lagarde P , Delespaul L , Lesluyes T , Le Guellec S , Pérot G , Leroy L , Coindre JM , Chibon F . Genome remodeling upon mesenchymal tumor cell fusion contributes to tumor progression and metastatic spread. Oncogene. 2020; 39:4198–211. 10.1038/s41388-020-1276-6. 32242148

[71] Miroshnychenko D , Baratchart E , Ferrall-Fairbanks MC , Velde RV , Laurie MA , Bui MM , Tan AC , Altrock PM , Basanta D , Marusyk A . Spontaneous cell fusions as a mechanism of parasexual recombination in tumour cell populations. Nat Ecol Evol. 2021; 5:379–91. 10.1038/s41559-020-01367-y. 33462489

[72] Duelli DM , Hearn S , Myers MP , Lazebnik Y . A primate virus generates transformed human cells by fusion. J Cell Biol. 2005; 171:493–503. 10.1083/jcb.200507069. 16275753 PMC2171256

[73] Parris GE . The role of viruses in cell fusion and its importance to evolution, invasion and metastasis of cancer clones. Med Hypotheses. 2005; 64:1011–14. 10.1016/j.mehy.2004.11.012. 15780502

[74] Duelli D , Lazebnik Y . Cell-to-cell fusion as a link between viruses and cancer. Nat Rev Cancer. 2007; 7:968–76. 10.1038/nrc2272. 18034186

[75] Saini G , Aneja R . Cancer as a prospective sequela of long COVID-19. Bioessays. 2021; 43:e2000331. 10.1002/bies.202000331. 33914346 PMC8206711

[76] van Doremalen N , Lambe T , Spencer A , Belij-Rammerstorfer S , Purushotham JN , Port JR , Avanzato VA , Bushmaker T , Flaxman A , Ulaszewska M , Feldmann F , Allen ER , Sharpe H , et al. ChAdOx1 nCoV-19 vaccine prevents SARS-CoV-2 pneumonia in rhesus macaques. Nature. 2020; 586:578–82. 10.1038/s41586-020-2608-y. 32731258 PMC8436420

[77] Bos R , Rutten L , van der Lubbe JEM , Bakkers MJG , Hardenberg G , Wegmann F , Zuijdgeest D , de Wilde AH , Koornneef A , Verwilligen A , van Manen D , Kwaks T , Vogels R , et al. Ad26 vector-based COVID-19 vaccine encoding a prefusion-stabilized SARS-CoV-2 Spike immunogen induces potent humoral and cellular immune responses. NPJ Vaccines. 2020; 5:91. 10.1038/s41541-020-00243-x. 33083026 PMC7522255

[78] Vogel AB , Kanevsky I , Che Y , Swanson KA , Muik A , Vormehr M , Kranz LM , Walzer KC , Hein S , Güler A , Loschko J , Maddur MS , Ota-Setlik A , et al. BNT162b vaccines protect rhesus macaques from SARS-CoV-2. Nature. 2021; 592:283–89. 10.1038/s41586-021-03275-y. 33524990

[79] Corbett KS , Edwards DK , Leist SR , Abiona OM , Boyoglu-Barnum S , Gillespie RA , Himansu S , Schäfer A , Ziwawo CT , DiPiazza AT , Dinnon KH , Elbashir SM , Shaw CA , et al. SARS-CoV-2 mRNA vaccine design enabled by prototype pathogen preparedness. Nature. 2020; 586:567–71. 10.1038/s41586-020-2622-0. 32756549 PMC7581537

[80] Rzymski P , Perek B , Flisiak R . Thrombotic Thrombocytopenia after COVID-19 Vaccination: In Search of the Underlying Mechanism. Vaccines (Basel). 2021; 9:559. 10.3390/vaccines9060559. 34071883 PMC8227748

[81] Ou X , Liu Y , Lei X , Li P , Mi D , Ren L , Guo L , Guo R , Chen T , Hu J , Xiang Z , Mu Z , Chen X , et al. Characterization of spike glycoprotein of SARS-CoV-2 on virus entry and its immune cross-reactivity with SARS-CoV. Nat Commun. 2020; 11:1620. 10.1038/s41467-020-15562-9. 32221306 PMC7100515

[82] Nguyen HT , Zhang S , Wang Q , Anang S , Wang J , Ding H , Kappes JC , Sodroski J . Spike Glycoprotein and Host Cell Determinants of SARS-CoV-2 Entry and Cytopathic Effects. J Virol. 2021; 95. 10.1128/JVI.02304-20. PMC8092844 33310888

[83] Cari L , Fiore P , Naghavi Alhosseini M , Sava G , Nocentini G . Blood clots and bleeding events following BNT162b2 and ChAdOx1 nCoV-19 vaccine: An analysis of European data. J Autoimmun. 2021; 122:102685. 10.1016/j.jaut.2021.102685. 34174723 PMC8220408

[84] Kragholm K , Sessa M , Mulvad T , Andersen MP , Collatz-Christensen H , Blomberg SN , Lippert F , Mikkelsen S , Leutscher P , Melgaard D , Torp-Pedersen C , Kristensen SR , Larsen TB , Sogaard P . Thrombocytopenia after COVID-19 vaccination. J Autoimmun. 2021; 123:102712. 10.1016/j.jaut.2021.102712. 34332437 PMC8313538

[85] Østergaard SD , Schmidt M , Horváth-Puhó E , Thomsen RW , Sørensen HT . Thromboembolism and the Oxford-AstraZeneca COVID-19 vaccine: side-effect or coincidence? Lancet. 2021; 397:1441–43. 10.1016/S0140-6736(21)00762-5. 33798498 PMC8009607

[86] Pottegård A , Lund LC , Karlstad Ø , Dahl J , Andersen M , Hallas J , Lidegaard Ø , Tapia G , Gulseth HL , Ruiz PL , Watle SV , Mikkel-sen AP , Pedersen L , et al. Arterial events, venous thromboembolism, thrombocytopenia, and bleeding after vaccination with Oxford-AstraZeneca ChAdOx1-S in Denmark and Norway: population based cohort study. BMJ. 2021; 373:n1114. 10.1136/bmj.n1114. 33952445 PMC8097496

[87] Oxford-AstraZeneca COVID-19 vaccine. Wikipedia. 2021. https://en.wikipedia.org/w/index.php?title=Oxford%E2%80%93AstraZeneca_COVID-19_vaccine&oldid=1029219093.

[88] Greinacher A , Thiele T , Warkentin TE , Weisser K , Kyrle PA , Eichinger S . Thrombotic Thrombocytopenia after ChAdOx1 nCov-19 Vaccination. N Engl J Med. 2021. 10.1056/NEJMoa2104840. PMC8095372 33835769

[89] Baker AT , Boyd RJ , Sarkar D , Vant J , Crespo AT , Waraich K , Truong CD , Bates E , Wilson E , Chan CK , Lipka-Lloyd M , Fromme P , Nagalo MB , et al. The Structure of ChAdOx1/AZD-1222 Reveals Interactions with CAR and PF4 with Implications for Vaccine-induced Immune Thrombotic Thrombocytopenia. bioRxiv. 2021; 2021.05.19.444882. 10.1101/2021.05.19.444882.

[90] Kowarz E , Krutzke L , Reis J , Bracharz S , Kochanek S , Marschalek R . “Vaccine-Induced Covid-19 Mimicry” Syndrome:Splice reactions within the SARS-CoV-2 Spike open reading frame result in Spike protein variants that may cause thromboembolic events in patients immunized with vector-based vaccines. In Review; 2021. Available from https://www.researchsquare.com/article/rs-558954/v1.

[91] Taquet M , Husain M , Geddes JR , Luciano S , Harrison PJ . Cerebral venous thrombosis and portal vein thrombosis: A retrospective cohort study of 537,913 COVID-19 cases. EClinicalMedicine. 2021; 39:101061. 10.1016/j.eclinm.2021.101061. 34368663 PMC8324974

[92] Watanabe Y , Mendonça L , Allen ER , Howe A , Lee M , Allen JD , Chawla H , Pulido D , Donnellan F , Davies H , Ulaszewska M , Belij-Rammerstorfer S , Morris S , et al. Native-like SARS-CoV-2 Spike Glycoprotein Expressed by ChAdOx1 nCoV-19/AZD1222 Vaccine. ACS Cent Sci. 2021; 7:594–602. 10.1021/acscentsci.1c00080. 34056089 PMC8043200

[93] Almuqrin A , Davidson AD , Williamson MK , Lewis PA , Heesom KJ , Morris S , Gilbert SC , Matthews DA . SARS-CoV-2 vaccine ChAdOx1 nCoV-19 infection of human cell lines reveals low levels of viral backbone gene transcription alongside very high levels of SARS-CoV-2 S glycoprotein gene transcription. Genome Med. 2021; 13:43. 10.1186/s13073-021-00859-1. 33722288 PMC7958140

[94] Pallesen J , Wang N , Corbett KS , Wrapp D , Kirchdoerfer RN , Turner HL , Cottrell CA , Becker MM , Wang L , Shi W , Kong WP , Andres EL , Kettenbach AN , et al. Immunogenicity and structures of a rationally designed prefusion MERS-CoV spike antigen. Proc Natl Acad Sci U S A. 2017; 114:E7348–57. 10.1073/pnas.1707304114. 28807998 PMC5584442

[95] Kirchdoerfer RN , Wang N , Pallesen J , Wrapp D , Turner HL , Cottrell CA , Corbett KS , Graham BS , McLellan JS , Ward AB . Stabilized coronavirus spikes are resistant to conformational changes induced by receptor recognition or proteolysis. Sci Rep. 2018; 8:15701. 10.1038/s41598-018-34171-7. 30356097 PMC6200764

[96] Xia S , Lan Q , Su S , Wang X , Xu W , Liu Z , Zhu Y , Wang Q , Lu L , Jiang S . The role of furin cleavage site in SARS-CoV-2 spike protein-mediated membrane fusion in the presence or absence of trypsin. Signal Transduct Target Ther. 2020; 5:92. 10.1038/s41392-020-0184-0. 32532959 PMC7289711

[97] Fuentes-Prior P . Priming of SARS-CoV-2 S protein by several membrane-bound serine proteinases could explain enhanced viral infectivity and systemic COVID-19 infection. J Biol Chem. 2021; 296:100135. 10.1074/jbc.REV120.015980. 33268377 PMC7834812

[98] Hörnich BF , Großkopf AK , Schlagowski S , Tenbusch M , Kleine-Weber H , Neipel F , Stahl-Hennig C , Hahn AS . SARS-CoV-2 and SARS-CoV Spike-Mediated Cell-Cell Fusion Differ in Their Requirements for Receptor Expression and Proteolytic Activation. J Virol. 2021; 95:e00002–21. 10.1128/JVI.00002-21. 33608407 PMC8104116

[99] Koppisetti RK , Fulcher YG , Van Doren SR . Fusion Peptide of SARS-CoV-2 Spike Rearranges into a Wedge Inserted in Bilayered Micelles. J Am Chem Soc. 2021; 143:13205–11. 10.1021/jacs.1c05435. 34375093 PMC8370118

[100] Logunov DY , Dolzhikova IV , Shcheblyakov DV , Tukhvatulin AI , Zubkova OV , Dzharullaeva AS , Kovyrshina AV , Lubenets NL , Grousova DM , Erokhova AS , Botikov AG , Izhaeva FM , Popova O , et al. Safety and efficacy of an rAd26 and rAd5 vector-based heterologous prime-boost COVID-19 vaccine: an interim analysis of a randomised controlled phase 3 trial in Russia. The Lancet. 2021; 397:671–81. 10.1016/S0140-6736(21)00234-8. 33545094 PMC7852454

[101] Kory P , Meduri GU , Varon J , Iglesias J , Marik PE . Review of the Emerging Evidence Demonstrating the Efficacy of Ivermectin in the Prophylaxis and Treatment of COVID-19. Am J Ther. 2021; 28:e299–318. 10.1097/MJT.0000000000001377. 34375047 PMC8088823

[102] Crump A . Ivermectin: enigmatic multifaceted 'wonder' drug continues to surprise and exceed expectations. J Antibiot (Tokyo). 2017; 70:495–505. 10.1038/ja.2017.11. 28196978

[103] Zang J , Zhu Y , Zhou Y , Gu C , Yi Y , Wang S , Xu S , Hu G , Du S , Yin Y , Wang Y , Yang Y , Zhang X , et al. Yeast-produced RBD-based recombinant protein vaccines elicit broadly neutralizing antibodies and durable protective immunity against SARS-CoV-2 infection. Cell Discov. 2021; 7:71. 10.1038/s41421-021-00315-9. 34408130 PMC8372230

[104] Wang H , Zhang Y , Huang B , Deng W , Quan Y , Wang W , Xu W , Zhao Y , Li N , Zhang J , Liang H , Bao L , Xu Y , et al. Development of an Inactivated Vaccine Candidate, BBIBP-CorV, with Potent Protection against SARS-CoV-2. Cell. 2020; 182:713–21.e9. 10.1016/j.cell.2020.06.008. 32778225 PMC7275151

[105] Gao Q , Bao L , Mao H , Wang L , Xu K , Yang M , Li Y , Zhu L , Wang N , Lv Z , Gao H , Ge X , Kan B , et al. Development of an inactivated vaccine candidate for SARS-CoV-2. Science. 2020; 369:77–81. 10.1126/science.abc1932. 32376603 PMC7202686

[106] Al Kaabi N , Zhang Y , Xia S , Yang Y , Al Qahtani MM , Abdulrazzaq N , Al Nusair M , Hassany M , Jawad JS , Abdalla J , Hussein SE , Al Mazrouei SK , Al Karam M , et al. Effect of 2 Inactivated SARS-CoV-2 Vaccines on Symptomatic COVID-19 Infection in Adults: A Randomized Clinical Trial. JAMA. 2021; 326:35–45. 10.1001/jama.2021.8565. 34037666 PMC8156175

[107] Liu T , Dai J , Yang Z , Yu X , Xu Y , Shi X , Wei D , Tang Z , Xu G , Xu W , Liu Y , Shi C , Ni Q , et al. Inactivated SARS-CoV-2 vaccine does not influence the profile of prothrombotic antibody nor increase the risk of thrombosis in a prospective Chinese cohort. Sci Bull (Beijing). 2021; 66:2312–19. 10.1016/j.scib.2021.07.033. 34336365 PMC8313791

[108] Vaccine Adverse Event Reporting System (VAERS). Available 2021 Aug 5, from https://vaers.hhs.gov/.

[109] European database of suspected adverse drug reaction reports. https://www.adrreports.eu/en/index.html.

[110] https://www.modernatx.com/pipeline.

[111] Gürtler L , Seitz R , Schramm W . Cerebral venous thrombosis after COVID-19 vaccination: is the risk of thrombosis increased by intravascular application of the vaccine? Infection. 2021; 49:1071–74. 10.1007/s15010-021-01658-x. 34286453 PMC8294245

[112] Zhang S , Liu Y , Wang X , Yang L , Li H , Wang Y , Liu M , Zhao X , Xie Y , Yang Y , Zhang S , Fan Z , Dong J , et al. SARS-CoV-2 binds platelet ACE2 to enhance thrombosis in COVID-19. J Hematol Oncol. 2020; 13:120. 10.1186/s13045-020-00954-7. 32887634 PMC7471641

[113] Chen L , Li X , Chen M , Feng Y , Xiong C . The ACE2 expression in human heart indicates new potential mechanism of heart injury among patients infected with SARS-CoV-2. Cardiovasc Res. 2020; 116:1097–100. 10.1093/cvr/cvaa078. 32227090 PMC7184507

[114] Sharma A , Li X , Bangari DS , Mittal SK . Adenovirus receptors and their implications in gene delivery. Virus Res. 2009; 143:184–94. 10.1016/j.virusres.2009.02.010. 19647886 PMC2903974

[115] Verbeke R , Lentacker I , De Smedt SC , Dewitte H . The dawn of mRNA vaccines: The COVID-19 case. J Control Release. 2021; 333:511–20. 10.1016/j.jconrel.2021.03.043. 33798667 PMC8008785

